# Phase Ib Study of Atezolizumab Plus Interferon-α with or without Bevacizumab in Patients with Metastatic Renal Cell Carcinoma and Other Solid Tumors

**DOI:** 10.3390/curroncol28060455

**Published:** 2021-12-20

**Authors:** Christian U. Blank, Deborah J. Wong, Thai H. Ho, Todd M. Bauer, Carrie B. Lee, Fabiola Bene-Tchaleu, Jing Zhu, Xiaosong Zhang, Edward Cha, Mario Sznol

**Affiliations:** 1 Netherlands Cancer Institute-Antoni van Leeuwenhoek, 1066 Amsterdam, The Netherlands; c.blank@nki.nl; 2 David Geffen School of Medicine, University of California Los Angeles, Los Angeles, CA 90095, USA; DeWong@mednet.ucla.edu; 3 Division of Hematology and Medical Oncology, Mayo Clinic, Scottsdale, AZ 85259, USA; ho.thai@mayo.edu; 4 Sarah Cannon Research Institute/Tennessee Oncology, PLLC, Nashville, TN 37203, USA; tbauer@tnonc.com; 5 Division of Hematology/Oncology, University of North Carolina at Chapel Hill, Chapel Hill, NC 27599, USA; carrie_lee@med.unc.edu; 6 Hoffmann-La Roche Ltd., Mississauga, ON L5N 5M8, Canada; fabiola.bene_tchaleu@roche.com; 7 Genentech, Inc., South San Francisco, CA 94080, USA; zhu.jing@gene.com (J.Z.); susanzhang888@gmail.com (X.Z.); cha.edward@gene.com (E.C.); 8 Yale Cancer Center, Yale School of Medicine, New Haven, CT 06520, USA

**Keywords:** atezolizumab, bevacizumab, checkpoint inhibition, interferon alfa, renal cell carcinoma

## Abstract

This Phase Ib study combined programmed death-ligand 1 inhibitor, atezolizumab, with other immunomodulatory agents in locally advanced and metastatic solid tumors. Arms B-D evaluated atezolizumab plus interferon-α, with/without vascular endothelial growth factor inhibitor, bevacizumab, in renal cell carcinoma (RCC) and other solid tumors. Arm B predominantly recruited patients with previously treated RCC or melanoma to receive atezolizumab plus interferon α-2b. Arm C investigated atezolizumab plus polyethylene glycol (PEG)-interferon α-2a in previously treated RCC. Arm D evaluated atezolizumab plus PEG-interferon α-2a and bevacizumab. Primary objectives were safety and tolerability; secondary objectives included clinical activity. Combination therapy was well tolerated, with safety profiles consistent with known risks of individual agents. The most frequent treatment-related toxicities were fatigue, chills, and pyrexia. The objective response rate (ORR) in arm B was 20.0% overall and 17.8% in patients with previously treated checkpoint inhibitor–naive RCC (*n* = 45). No responses were reported in arm C. The highest ORR in arm D was 46.7% in patients with treatment-naive RCC (*n* = 15). Data showed preliminary clinical activity and acceptable tolerability of atezolizumab plus interferon α-2b in patients with previously treated checkpoint inhibitor–naive RCC and of atezolizumab plus PEG-interferon α-2a and bevacizumab in patients with treatment-naive RCC.

## 1. Introduction

Immunotherapy, in the form of interleukin 2 and interferon-α, has been used to treat cancers, such as metastatic renal cell carcinoma (RCC) and melanoma since the early 1990s [1,2]. More recently, immune-checkpoint blockade targeting cytotoxic T-lymphocyte-associated protein 4 (CTLA-4), programmed death-ligand 1 (PD-L1), or programmed death-1 (PD-1) improved survival [3,4,5,6,7,8,9,10] in both of these tumor types. In first-line treatment of advanced or metastatic RCC, combinations of the checkpoint inhibitors (CPI) ipilimumab (anti–CTLA-4) and nivolumab (anti–PD-1), and anti–PD-L1 or anti–PD-1 in combination with anti-angiogenic agents, improved outcomes compared with the anti-angiogenic agent sunitinib in randomized phase III trials [6,7,10,11,12]. Novel therapies are required for the high proportion of patients who do not respond to treatment or relapse [2,13]. In this five-arm Phase Ib study, atezolizumab was combined with other immunomodulatory therapies in patients with various locally advanced or metastatic solid tumors.

Although interferon α-2a was the first immunotherapy agent to be approved for the treatment of cancer, the mechanisms of its anti-tumor effects are not yet fully understood [14]. Interferons induce major histocompatibility complex upregulation and subsequent antigen presentation [15] and promote tumor PD-L1 expression, potentially weakening T cell responses in the tumor microenvironment [16,17], thus providing a rationale for combining interferons with antagonists of the PD-1/PD-L1 pathway. An initial Phase Ib/II study of pembrolizumab plus polyethylene glycol (PEG) interferon produced anti-tumor responses in patients with advanced melanoma [18].

Bevacizumab is a recombinant, humanized therapeutic antibody against vascular endothelial growth factor (VEGF) and, therefore, inhibits tumor angiogenesis. Bevacizumab was previously approved for use in RCC in combination with interferon α-2a based on significant progression-free survival (PFS) improvement versus interferon α-2a alone [19]. Because VEGF also inhibits dendritic cell maturation and promotes cancer immune evasion through multiple mechanisms [20,21,22], bevacizumab may produce beneficial immune modulatory effects in patients with advanced cancer. A Phase I study of bevacizumab and atezolizumab demonstrated that bevacizumab increased chemokine production and T cell traffic in tumors [23]. In a Phase II study, bevacizumab improved response to atezolizumab in patients with RCC whose tumors expressed both a T cell effector gene signature and a suppressive myeloid cell signature [24]. In the IMmotion151 Phase III trial, bevacizumab plus atezolizumab improved PFS compared with sunitinib in the intention-to-treat population of previously untreated patients with RCC unselected for PD-L1 expression [25]. 

We hypothesized that atezolizumab plus bevacizumab plus PEG-interferon α-2a could further increase the anti-tumor immune response and provide a more durable clinical benefit. Because chronic exposure to interferon-α may increase the likelihood of experiencing toxicity, additionally suppress immune responses [26], and cause rapid desensitization to repeat dosing [27], the study was designed to administer interferon for only a short duration. In later cohorts, standard interferon-α was replaced with PEG-interferon α-2a, which can be administered weekly and is approved for the treatment of hepatitis C, for which it demonstrated improved efficacy over interferon α-2a [28,29].

This manuscript presents data from arms B-D, which each investigated the safety and clinical activity of atezolizumab in combination with an abbreviated course of interferon-α (interferon α-2b or PEG-interferon α-2a, with or without bevacizumab), in patients with advanced solid tumors, predominantly RCC. 

## 2. Materials and Methods

### 2.1. Study Design

This was an international, open-label, multicenter, non-randomized Phase Ib trial investigating the combination of atezolizumab with other immunomodulatory agents (NCT02174172). Five study arms enrolled patients with specific tumor types to receive atezolizumab with different combination partners. Arm A combined atezolizumab with ipilimumab, and arm E evaluated obinutuzumab with atezolizumab, which are not reported. Data from arms B, C, and D of the study are reported here.

The primary objectives of arm B were used to evaluate the safety and tolerability of atezolizumab plus interferon α-2b in patients with RCC or melanoma and to identify a recommended Phase II dose and schedule for this combination. 

The primary objectives of arm C were to evaluate the safety and tolerability of atezolizumab plus PEG-interferon α-2a in patients with advanced or metastatic RCC. The opening of arm D occurred only upon completion of a 21-day safety evaluation period for patients enrolled in arm C. The primary objectives of arm D were to evaluate the safety and tolerability of atezolizumab plus PEG-interferon α-2a and bevacizumab in patients with advanced or metastatic solid tumors. 

### 2.2. Patients

Eligible patients were aged ≥18 years, had a baseline Eastern Cooperative Oncology Group (ECOG) performance status of 0 or 1, a measurable disease at baseline according to Response Evaluation Criteria in Solid Tumours version 1.1 (RECIST 1.1), and adequate hematologic and organ function. Patients with active or untreated central nervous system (CNS) metastases were ineligible for participation; treated CNS metastases were permitted if no ongoing corticosteroid treatment was required and if there was no evidence of disease progression from the time of completion of CNS-directed therapy until study screening.

Patients had to have histologically or cytologically documented locally advanced or metastatic solid tumors of a type eligible for the specific cohort. Those with actionable alterations in *EGFR* or *ALK* for non-small cell lung cancer (NSCLC) or *BRAF* for melanoma had to have previously failed or be intolerant to the relevant targeted therapies. All patients had to have archival tumor tissue available in a representative formalin-fixed paraffin-embedded tumor specimen collected at first diagnosis and/or subsequent recurrences and an associated pathology report. Additional criteria for individual arms are included in the Supplemental Material and Methods (Supplementary Table S1). 

### 2.3. Treatment

All arms evaluated atezolizumab plus interferon-α, with or without bevacizumab. In arm B, patients in the dose-escalation stage were treated as shown in Supplementary Figure S1 (Supplementary Materials and Methods). Following completion of the dose-escalation stage, the expansion stage of arm B was enrolled at the maximum tolerated dose (MTD) or the highest tolerated dose level tested if the MTD was not identified. Patients in arms C and D received IV atezolizumab 1200 mg every 3 weeks (q3w) and 6 cycles of SC PEG-interferon α-2a 180 μg q3w. Additionally, patients in arm D received IV bevacizumab 15 mg/kg q3w.

Atezolizumab (in all study arms) and bevacizumab (arm D) could be continued until loss of clinical benefit, determined by the investigator after assessment of radiographic data, biopsy results, and the patient’s clinical status.

### 2.4. Endpoints and Assessments

Safety outcome measures across all study arms were the nature and frequency of dose-limiting toxicities; the nature, frequency, and severity of adverse events (AEs); and changes in vital signs, physical findings, and laboratory study results during and after atezolizumab administration. AE assessment occurred on days 1, 8, and 15 of cycle 1 and day 1 of subsequent cycles, and grading was performed according to the National Cancer Institute Common Terminology Criteria for Adverse Events version 4.0. AEs of special interest were pre-defined in the protocol and included those considered to be associated with each investigational agent.

The secondary efficacy endpoints were PFS, objective response rate (ORR; sum of confirmed partial responses (PR) and complete responses (CR)), best overall response, duration of objective response (DOR), and overall survival (OS). Additional details on statistical analyses are provided in the Supplemental Methods.

## 3. Results

### 3.1. Patients

**Figure 1:**
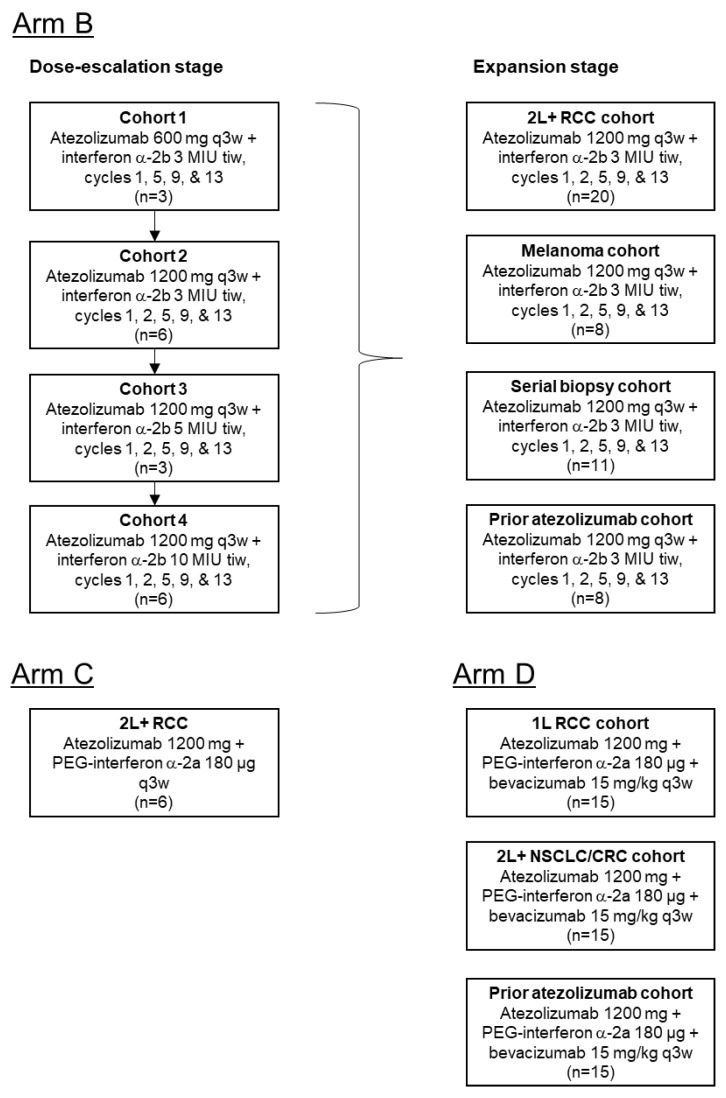
Patient disposition. A total of 116 patients were enrolled into Arms B, C, and D of the study. Arm B recruited 65 patients with previously treated RCC or melanoma into 4 cohorts, with each cohort receiving different dosing regimens of atezolizumab + interferon-a-2b in the initial dose-escalation stage. At the dose expansion stage, patients in Arm B received atezolizumab 1200 mg q3w + interferon a-2b 3 MIU tiw. Arm C comprised 6 patients with previously treated RCC, and they received atezolizumab 1200 mg + PEG-interferon a-2a 180 µg q3w. Arm D recruited 45 patients into 3 cohorts, with each patient receiving atezolizumab 1200 mg + PEG-interferon a-2a 180 µg + bevacizumab 15 mg/kg q3w. MIU, million international units; q3w, every 3 weeks; tiw, 3 times per week.

In total, 116 patients were enrolled between August 2014–October 2017 (Arm B: 65; Arm C: 6; Arm D: 45), comprising the safety and efficacy analysis populations for each arm (Figure 1). Reasons for discontinuation from the study are shown in Supplementary Table S2.

The majority of patients were white (84.5%), male (76.7%), had an ECOG performance status of 0 (72.4%), and did not have detectable PD-L1 expression on ≥1% of tumor-infiltrating immune cells (46–67%) or tumor cells (62–83%) (Table 1).

### 3.2. Dose Escalation

In arm B, the only dose-limiting toxicity reported during dose escalation was grade 4 hyperglycemia lasting 1 day in one patient enrolled in cohort 2 (atezolizumab 1200 mg q3w plus interferon α-2b 3 million international units (MIU) 3 times/week). The MTD for atezolizumab with interferon α-2b was not reached. Based on data across dose-escalation cohorts, the cohort 2 regimen (interferon α-2b 3 MIU on days 1, 3, 5, 8, 10, and 12 of cycle 1, plus atezolizumab 1200 mg on day 8 of cycle 1 and day 1 of subsequent cycles) was selected for dose expansion.

### 3.3. Safety and Activity: Arm B (Atezolizumab plus Interferon α-2b)

Arm B comprised 50 (76.9%) patients with previously treated RCC, 14 (21.5%) patients with melanoma, and 1 (1.5%) patient with NSCLC. Of the patients with RCC, 45 had not previously received treatment with a CPI. Information on tumor histology for patients with RCC is presented in Supplementary Table S3. In arm B, the median duration of therapy (range) with atezolizumab and interferon α-2b was 5.0 (0–59) months and 3.2 (0–9) months, respectively (Supplementary Table S4). 

All patients in arm B experienced ≥1 treatment-emergent AE of any grade (Table 2). The most frequently reported treatment-emergent AEs were fatigue (53.8%), chills (43.1%), and pyrexia (40.0%). The most common AEs related to atezolizumab were fatigue (27.7%), chills (18.5%), and arthralgia (11 patients, 16.9%), and the most common AEs related to interferon α-2b were chills (41.5%), fatigue (38.5%), and pyrexia (35.4%) (Table 3). Grade ≥3 treatment-emergent AEs were seen in 25 (38.5%) patients in arm B (Table 2), with the most common being anemia and pneumonia (7.7% each) and increased lipase (6.2%) (Supplementary Table S5).

Three (4.6%) patients in arm B discontinued atezolizumab or interferon α-2b because of AEs (Table 2). AEs resulted in interruption of atezolizumab in 13 (20.0%) patients in arm B, with the most common being anemia (4.6%) and arthralgia (3.1%). Interferon α-2b dose modification or interruption occurred in seven (10.8%) patients (Table 2). Grade 5 events were reported in three patients in arm B, which were all unrelated to treatment.

**Figure 2:**
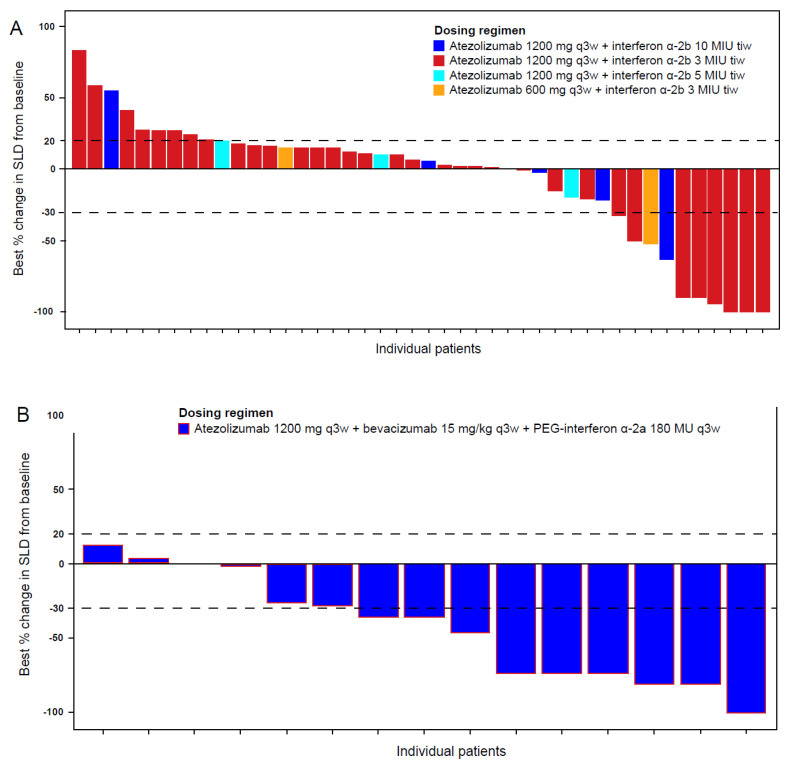
Best percent changes from baseline in target lesion size for individual patients. (**A**), Best percentage change from baseline in SLD in Arm B patients with RCC and no prior CPI. (**B**), Best percentage change from baseline in SLD in Arm D Cohort 1 patients with previously untreated RCC. Dashed lines indicate +20% and −30% values use to categorize RECIST response status. MIU, million international units; q3w, every 3 weeks; SLD, sum of longest diameters; tiw, three times per week.

Confirmed responses were achieved in 13 (20.0%) patients overall in arm B, 1 of which was a CR (Table 4). In the subgroup of patients with CPI-naive RCC, eight (17.8%) patients responded to treatment, with one CR and seven PRs. Changes from baseline in target lesion size are shown in Figure 2A. One patient (20.0%) with RCC and prior CPI treatment achieved a PR (Supplementary Table S6). The median DOR (95% CI) in arm B was 28.8 (16.8-NE) months overall and 24.9 (3.7-NE) months in CPI-naive RCC (Table 4). The median follow-up duration (range) in arm B was 38.8 (0.7–58.8) months. Median PFS (95% CI) was 4.1 months (3.0–5.5) overall and 3.2 months (2.8–5.5) in the CPI-naive RCC subgroup; 1-year PFS estimates were 24.6% and 20.0%, respectively (Table 4). Median OS (95% CI) was 29.9 (21.9–41.9) months overall and 26.3 (15.6–37.6) months in the CPI-naive RCC subgroup.

### 3.4. Safety and Activity: Arm C (Atezolizumab Plus PEG-Interferon α-2a)

All six patients in arm C had RCC and had received previous systemic therapy; tumor histology is presented in Supplementary Table S3. In this arm, the median duration of therapy (range) with atezolizumab and PEG-interferon α-2a was 5.2 (1–26) months and 2.8 (1–3) months, respectively (Supplementary Table S4). 

All patients in arm C experienced ≥1 treatment-emergent AE of any grade (Table 2). The most frequently reported treatment-emergent AEs in arm C were fatigue (50.0%), and anemia, anxiety, vomiting, decreased appetite, and nausea (33.3% each). The most common AE related to therapy for both atezolizumab and PEG-interferon α-2a was fatigue (33.3%) (Table 3). Grade 3 treatment-emergent AEs were seen in three (50.0%) patients (Supplementary Table S7); no grade 4 or 5 AEs were reported.

One (16.7%) patient in arm C discontinued atezolizumab and PEG-interferon α-2a because of grade 3 myasthenia gravis. Two (33.3%) patients experienced AEs, resulting in interruption of atezolizumab, while one patient required dose modification or interruption of PEG-interferon α-2a (Table 2).

No responses were seen in arm C; two of five evaluable patients had a stable disease as their best confirmed response (Table 4). The median follow-up duration (range) in arm C was 23.8 (1.2–26.0) months. All patients experienced PFS events within 9 months of starting treatment; median PFS was 1.9 months (95% CI 1.2–4.2), but OS data were not mature.

### 3.5. Safety and Activity: Arm D (Atezolizumab Plus PEG-Interferon α-2a and Bevacizumab)

Arm D cohort 1 included 15 (100%) patients with RCC, cohort 2 contained 14 (93.3%) patients with CRC and 1 (6.7%) patient with NSCLC, and cohort 3 comprised 6 (40.0%) patients with RCC and NSCLC and 3 (20.0%) patients with melanoma. Information on tumor histology for patients with RCC is presented in Supplementary Table S3. 

The median duration of therapy (range) with atezolizumab was 10.6 (2–30) months, 3.5 (1–26) months, and 5.6 (0–28) months in cohorts 1, 2, and 3, respectively (Supplementary Table S8).

All patients in arm D experienced ≥1 treatment-emergent AE of any grade (Table 2). The most frequently reported treatment-emergent AEs were fatigue (71.1%), headache (35.6%), and proteinuria (31.1%).

The most common AEs related to each component of treatment were fatigue (51.1%), pyrexia (15.6%), and chills, myalgia, and nausea (13.3% each) for atezolizumab; fatigue (55.6%), influenza-like illness (20.0%), and arthralgia and pyrexia (17.8% each) for PEG-interferon α-2a; and fatigue (35.6%), proteinuria (26.7%), and hypertension (22.2%) for bevacizumab (Table 3). Grade ≥3 treatment-emergent AEs were seen in 28 (62.2%) arm D patients (Table 2). The most common were hypertension (13.3%) and increased lipase (8.9%) (Supplementary Table S9).

One (2.2%) patient in arm D discontinued atezolizumab because of an AE and eight (17.8%) discontinued bevacizumab), while two patients (4.4%) discontinued PEG-interferon α-2a (Table 2). AEs resulted in interruption of atezolizumab in 7 (15.6%) patients, bevacizumab in 11 (24.4%) patients, and dose modification or interruption of PEG-interferon α-2a in 6 (13.3%) patients (Table 2).

One patient in cohort 3 of arm D experienced a Grade 5 AE related to bevacizumab (large intestine perforation).

In arm D, seven (46.7%) patients with previously untreated RCC in cohort 1 had confirmed responses, with one being a CR (Table 4; Supplementary Table S6). Changes from baseline in target lesion size are shown in Figure 2B. The median DOR (95% CI) in cohort 1 was 12.5 (4.5-NE) months. Two confirmed PRs each were reported in cohorts 2 and 3, including one PR in a patient with RCC (Table 4; Supplementary Table S6); median DOR was not evaluable in either cohort.

The median follow-up durations (range) in arm D were 17.3 (4.9–30.4) months in cohort 1, 19.7 (3.0+ to 26.3) months in cohort 2, and 18.2 (1.6+ to 27.6) months in cohort 3. Median PFS (95% CI) in cohorts 1, 2, and 3 was 9.0 months (4.1-NE), 3.2 months (2.5–4.3), and 6.9 months (4.4–8.4), respectively. The 1-year PFS estimates were 43.1%, 20.0%, and 17.8% (Table 4). Owing to limited numbers of events in some cohorts, OS data were mature only in cohort 2 (previously treated CRC or NSCLC), with a median (95% CI) of 12.7 months (5.5–19.9) (Table 4).

## 4. Discussion

This study investigated the combination of atezolizumab with interferon-α, in the form of interferon α-2b or PEG-interferon α-2a (with or without bevacizumab), in patients with advanced solid tumors. 

In arm B, atezolizumab plus interferon α-2b demonstrated preliminary clinical activity in melanoma and previously treated RCC, with durable clinical responses observed. Although no conclusions can be made, efficacy data in the largest group of patients in arm B, those with CPI-naive RCC, showed a similar median OS and numerically smaller median PFS and ORR compared with those for PD-1 inhibitor nivolumab alone in previously treated RCC [5]. This study investigated using a shorter course of interferon-α to reduce toxicity and potential suppression of immune responses. Although this strategy appears to have successfully mitigated the safety impact of combination therapy relative to atezolizumab alone [24,30], the lack of any efficacy benefit could be attributed to insufficient interferon dosing, rather than an absence of synergy between these agents.

Preliminary clinical activity of atezolizumab plus PEG-interferon α-2a and bevacizumab was detected in first-line RCC in arm D cohort 1. While the ORR was higher than that obtained in the intention-to-treat population of the IMmotion151 Phase III trial of atezolizumab plus bevacizumab in RCC (47% vs. 37%), median PFS was numerically shorter (9.0 vs. 11.2 months) [25]. Given the small number of patients, no definitive conclusion can be made. Similar data were obtained with axitinib plus avelumab, axitinib plus pembrolizumab, and ipilimumab plus nivolumab in populations from Phase III trials that were not selected for PD-L1 expression [6,7,10]. Combination regimens inhibiting both VEGF and PD-L1/PD-1 are active and approved for use in RCC [6,7,10]. Further, atezolizumab plus bevacizumab is the standard of care for patients with unresectable hepatocellular carcinoma [31], and atezolizumab plus bevacizumab in combination with chemotherapy is approved for patients with metastatic non-squamous NSCLC and no *EGFR* or *ALK* alterations [32]. Despite this evidence for activity, the addition of PEG-interferon α-2a to this type of combination does not substantially increase activity in RCC. 

Arm C contained few patients and was predominantly intended as a safety run-in for the atezolizumab plus PEG-interferon α-2 a combination. Some responses were detected in cohorts 2 and 3 of arm D, but the number is too small to draw meaningful conclusions. 

Generally, the combinations studied did not have improved activity over established regimens. Low prevalence of PD-L1 expression in this study population compared with rates previously reported [33] may have impacted clinical activity. Another main limitation of this study was the small number of patients included in each cohort. Sample sizes were selected to evaluate safety, pharmacokinetics, and pharmacodynamics and not powered for type I error purposes. The study also lacked comparator cohorts. Additional selection of subgroups by immune status or tumor types may have provided further insight into optimal interferon combination with other agents. While assessments of tumor vascular density and CD8+ cell infiltration are of interest, these parameters were beyond the scope of this study, but may be evaluated in future investigations. 

This study showed that atezolizumab, in combination with interferon α-2b or PEG-interferon α-2a with or without bevacizumab, was well tolerated. The safety profiles of the combination treatment regimens were consistent with the known risks of each individual study agent. It is unclear whether the addition of interferons contributed to efficacy. However, it is possible that the interferon regiment was not sufficient for an adequate immune response, which may have contributed to the lack of efficacy observed. Additional studies are warranted to optimize the dosing of interferon α-2b and PEG-interferon α-2a in combination with atezolizumab and bevacizumab to achieve efficacious results, while mitigating AEs. Although this could be attributed to the impact of limited dosing, it is also possible that blockade of additional signals may be needed to modulate immunity and overcome tumor resistance mechanisms. The safety profile of these interferon combinations suggests that the addition of other agents is possible but may be more effective in subgroups selected by immune status or in tumor types where bevacizumab and atezolizumab have proven biologic activity.

## 5. Conclusions

This Phase Ib study provided preliminary data on the clinical activity for atezolizumab with interferon α-2b in previously treated, CPI-naive RCC, atezolizumab plus PEG-interferon α-2a, and bevacizumab in previously untreated RCC. These combinations were well tolerated, but the inclusion of additional agents did not improve efficacy.

## Figures and Tables

**Table 1:** Baseline demographic and patient characteristics.

Characteristic	Arm B	Arm C	Arm D
	Atezo + IFNα	Atezo + PEG-IFNα	Atezo + PEG-IFNα + Bev
	(*n* = 65)	(*n* = 6)	(*n* = 45)
Tumor type, *n* (%)			
RCC	50 (76.9)	6 (100)	21 (46.7)
Melanoma	14 (21.5)	0	3 (6.7)
CRC	0	0	14 (31.1)
NSCLC	1 (1.5)	0	7 (15.6)
Age group, *n* (%)			
<65 y	39 (60.0)	2 (33.3)	30 (66.7)
≥65 y	26 (40.0)	4 (66.7)	15 (33.3)
Sex, *n* (%)			
Male	56 (86.2)	6 (100)	27 (60.0)
Female	9 (13.8)	0	18 (40.0)
Race, *n* (%)			
Black or African American	3 (4.6)	0	7 (15.6)
White	60 (92.3)	6 (100)	32 (71.1)
Asian	1 (1.5)	0	4 (8.9)
Unknown	1 (1.5)	0	2 (4.4)
ECOG performance status, *n* (%)			
0	49 (75.4)	3 (50.0)	32 (72.7) ^a^
1	16 (24.6)	3 (50.0)	12 (27.3) ^a^
Prior systemic therapy, *n* (%)			
Yes	55 (84.6)	6 (100)	30 (66.7)
No	10 (15.4)	0	15 (33.3)
Prior cancer surgery, *n* (%)			
Yes	61 (93.8)	6 (100)	26 (57.8)
No	4 (6.2)	0	19 (42.2)
Prior radiotherapy, *n* (%)			
Yes	36 (55.4)	3 (50.0)	16 (35.6)
No	29 (44.6)	3 (50.0)	29 (64.4)
PD-L1 IC score, *n* (%) ^b^			
IC0	30 (46.2)	4 (66.7)	27 (60.0)
IC1/2/3	26 (40.0)	1 (16.7)	3 (6.7)
Unknown	9 (13.8)	1 (16.7)	15 (33.3)
PD-L1 TC score, *n* (%) ^c^			
TC0	51 (78.5)	5 (83.3)	28 (62.2)
TC1/2/3	5 (7.7)	0	2 (4.4)
Unknown	9 (13.8)	1 (16.7)	15 (33.3)

atezo, atezolizumab; bev, bevacizumab; CRC, colorectal carcinoma; ECOG, Eastern Cooperative Oncology Group; IC, tumor-infiltrating immune cell; IFNα, interferon-α; NSCLC, non-small cell lung cancer; PEG-IFNα, polyethylene glycol interferon-; PD-L1, programmed death-ligand 1; RCC, renal cell carcinoma; TC, tumor cell. ^a^ Baseline ECOG PS was unavailable for 1 patient in Arm D. ^b^ Using VENTANA SP142 IHC assay (Ventana Medical Systems): IC0 was ≤ 1% PD-L1 expression on IC; IC1 was ≥ 1% and was <5% PD-L1 expression on IC; IC2 was ≥5% and was <10% PD-L1 expression on IC; IC3 was ≥ 10% PD-L1 expression on IC. ^c^ Using VENTANA SP142 IHC assay (Ventana Medical Systems): TC0 was < 1% PD-L1 expression on TC; TC1 was ≥ 1% and was <5% PD-L1 expression on TC; TC2 was ≥ 5% and was <50% PD-L1 expression on TC; TC3 was ≥ 50% PD-L1 expression on TC.

**Table 2:** Safety summary.

Patients with at Least 1, *n* (%)	Arm B	Arm C	Arm D
	Atezo + IFNα	Atezo + PEG-IFNα	Atezo + PEG-IFNα + Bev
	(*n* = 65)	(*n* = 6)	(*n* = 45)
Treatment-emergent AE of any grade	65 (100)	6 (100)	45 (100)
Treatment-emergent AE with fatal outcome	3 (4.6)	0	1 (2.2)
Serious treatment-emergent AE	19 (29.2)	4 (66.7)	19 (42.2)
Treatment-emergent Grade 3–5 AE	25 (38.5)	3 (50.0)	28 (62.2)
AE leading to drug withdrawal			
AE leading to atezo withdrawal	3 (4.6)	1 (16.7)	1 (2.2)
AE leading to IFNα withdrawal	3 (4.6)	–	–
AE leading to PEG-IFNα withdrawal	–	1 (16.7)	2 (4.4)
AE leading to bev withdrawal	–	–	8 (17.8)
AE leading to drug dose modification/interruption			
AE leading to atezo dose interruption	13 (20.0)	2 (33.3)	7 (15.6)
AE leading to IFNα dose modification/interruption	7 (10.8)	–	–
AE leading to PEG-IFNα dose modification/interruption	–	2 (33.3)	6 (13.3)
AE leading to bev dose interruption	–	–	11 (24.4)

AE, adverse event; atezo, atezolizumab; bev, bevacizumab; IFNα, interferon-α; PEG, polyethylene glycol.

**Table 3:** Most common treatment-related AEs of any grade (reported in ≥10% of patients in any arm).

AE, *n* (%)	Arm B		Arm C		Arm D		
	Atezo + IFNα		Atezo + PEG-IFNα		Atezo + PEG-IFNα + Bev		
	(*n* = 65)		(*n* = 6)		(*n* = 45)		
	Atezo	IFNα	Atezo	PEG-IFNα	Atezo	PEG-IFNα	Bev
Any treatment-related AE	44 (67.7)	60 (92.3)	6 (100)	6 (100)	38 (84.4)	38 (84.4)	39 (86.7)
Fatigue	18 (27.7)	25 (38.5)	2 (33.3)	2 (33.3)	23 (51.1)	25 (55.6)	16 (35.6)
Chills	12 (18.5)	27 (41.5)	0	0	6 (13.3)	6 (13.3)	5 (11.1)
Pyrexia	8 (12.3)	23 (35.4)	0	1 (16.7)	7 (15.6)	8 (17.8)	5 (11.1)
Arthralgia	11 (16.9)	9 (13.8)	0	0	4 (8.9)	8 (17.8)	1 (2.2)
Myalgia	7 (10.8)	14 (21.5)	0	0	6 (13.3)	4 (8.9)	2 (4.4)
Headache	4 (6.2)	6 (9.2)	1 (16.7)	1 (16.7)	5 (11.1)	5 (11.1)	5 (11.1)
Nausea	5 (7.7)	7 (10.8)	0	1 (16.7)	6 (13.3)	7 (15.6)	6 (13.3)
Influenza-like illness	3 (4.6)	8 (12.3)	0	0	5 (11.1)	9 (20.0)	4 (8.9)
Proteinuria	1 (1.5)	1 (1.5)	0	0	1 (2.2)	1 (2.2)	12 (26.7)
Hypertension	0	0	0	0	0	1 (2.2)	10 (22.2)
Hypothyroidism	8 (12.3)	2 (3.1)	1 (16.7)	0	5 (11.1)	1 (2.2)	0
Cough	2 (3.1)	2 (3.1)	1 (16.7)	1 (16.7)	1 (2.2)	2 (4.4)	1 (2.2)
Epistaxis	0	1 (1.5)	0	0	0	0	5 (11.1)
Eyelid ptosis	0	0	1 (16.7)	1 (16.7)	0	0	0
Blood creatinine phosphokinase increase	0	0	1 (16.7)	1 (16.7)	0	0	0
Transaminase increase	0	0	1 (16.7)	1 (16.7)	0	0	0
Decreased appetite	4 (6.2)	5 (7.7)	1 (16.7)	1 (16.7)	2 (4.4)	4 (8.9)	2 (4.4)
Myasthenia gravis	0	0	1 (16.7)	1 (16.7)	0	0	0
Acute respiratory failure	0	0	1 (16.7)	1 (16.7)	0	0	0
Hyperthyroidism	0	0	1 (16.7)	1 (16.7)	2 (4.4)	0	0
Vomiting	3 (4.6)	4 (6.2)	1 (16.7)	1 (16.7)	0	1 (2.2)	1 (2.2)
Muscular weakness	0	1 (1.5)	1 (16.7)	1 (16.7)	1 (2.2)	1 (2.2)	1 (2.2)
Pneumonia	0	0	1 (16.7)	1 (16.7)	0	0	0
Hypoxia	0	0	1 (16.7)	0	0	0	0
Respiratory acidosis	0	0	1 (16.7)	0	0	0	0
Tumor-associated fever	0	0	1 (16.7)	0	0	0	0
Anemia	3 (4.6)	5 (7.7)	0	1 (16.7)	3 (6.7)	3 (6.7)	2 (4.4)
Infusion-related reaction	1 (1.5)	0	0	1 (16.7)	1 (2.2)	0	0

AE, adverse event; atezo, atezolizumab; bev; bevacizumab; IFNα, interferon-α; PEG, polyethylene glycol.

**Table 4:** Efficacy summary.

	Arm B Atezo + IFNα		Arm C Atezo + PEG-IFNα (*n* = 6)	Arm D Atezo + PEG-IFNα + Bev		
	All Patients (*n* = 65)	CPI-Naive RCC (*n* = 45)		Cohort 1: 1L RCC (*n* = 15)	Cohort 2: 2L+ CRC/NSCLC (*n* = 15)	Cohort 3: Prior CPI (*n* = 15)
**Response, *n* (%) [95% CI]**						
Objective response rate	13 (20.0)	8 (17.8)	0	7 (46.7)	2 (13.3)	2 (13.3)
	[10.3–29.7]	[6.6–28.9]	[0.0–0.0]	[21.4–71.9]	[0.0–30.5]	[0.0–30.5]
Complete response	1 (1.5)	1 (2.2)	0	1 (6.7)	0	0
	[0.0–4.5]	[0.0–6.5]	[0.0–0.0]	[0.0–19.3]	[0.0–0.0]	[0.0–0.0]
Partial response	12 (18.5)	7 (15.6)	0	6 (40.0)	2 (13.3)	2 (13.3)
	[9.0–27.9]	[5.0–26.1]	[0.0–0.0]	[15.2–64.8]	[0.0–30.5]	[0.0–30.5]
Stable disease	30 (46.2)	20 (44.4)	2 (33.3)	7 (46.7)	8 (53.3)	11 (73.3)
	[34.0–58.3]	[29.9–59.0]	[0.0–71.0]	[21.4–71.9]	[28.1–78.6]	[50.9–95.7]
Progressive disease	20 (30.8)	16 (35.6)	3 (50.0)	1 (6.7)	5 (33.3)	2 (13.3)
	[19.6–42.0]	[21.6–49.5]	[10.0–90.0]	[0.0–19.3]	[9.5–57.2]	[0.0–30.5]
Missing/unevaluable	2 (3.1)	1 (2.2)	1 (16.7)	0	0	0
**Duration of response**	*n* = 13	*n* = 8	*n* = 0	*n* = 7	*n* = 2	*n* = 2
Median, month [95% CI]	28.8 [16.8-NE]	24.9 [3.7-NE]	–	12.5 [4.5-NE]	NE [11.3-NE]	NE [3.1-NE]
Range	2.8–51.8 ^a^	2.8–50.5 ^a^	–	2.8–25.8 ^a^	11.3–13.8 ^a^	3.1–19.4 ^a^
**Progression-free survival**						
Patients with event, *n* (%)	58 (89.2)	41 (91.1)	6 (100)	10 (66.7)	13 (86.7)	12 (80.0)
Median, month [95% CI]	4.1 [3.0–5.5]	3.2 [2.8–5.5]	1.9 [1.2–4.2]	9.0 [4.1-NE]	3.2 [2.5–4.3]	6.9 [4.4–8.4]
Range	1–57 ^a^	1–52 ^a^	1–9	1–28 ^a^	1–25 ^a^	1–28 ^a^
Landmark rate, % [95% CI]						
6 months	33.9 [22.3–45.4]	33.3 [19.6–47.1]	16.7 [0.0–46.5]	71.8 [48.3–95.3]	26.7 [4.3–49.0]	66.7 [42.8–90.5]
1 y	24.6 [14.1–35.1]	20.0 [8.3–31.7]	NE	43.1 [17.1–69.0]	20.0 [0.0–40.2]	17.8 [0.0–38.4]
2 y	13.3 [4.9–21.7]	10.4 [1.2–19.6]	NE	28.7 [5.0–52.4]	13.3 [0.0–30.5]	17.8 [0.0–38.4]
**Overall survival**						
Patients with event, *n* (%)	40 (61.5)	30 (66.7)	2 (33.3)	2 (13.3)	10 (66.7)	8 (53.3)
Median, month [95% CI]	29.9 [21.9–41.9]	26.3 [15.6–37.6]	NE [2.4-NE]	NE [17.7-NE]	12.7 [5.5–19.9]	13.9 [10.9-NE]
Range	1–59 ^a^	3 ^a^–53 ^a^	1 ^a^–26 ^a^	5 ^a^–30 ^a^	3–26 ^a^	2–28 ^a^
Landmark rate, % [95% CI]						
6 months	90.6 [83.4–97.8]	93.1 [85.5–100]	80.0 [44.9–100]	100 [100–100]	72.2 [49.0–95.4]	80.0 [59.8–100]
1 y	77.8 [67.6–88.1]	74.4 [61.3–87.4]	80.0 [44.9–100]	92.9 [79.4–100]	50.6 [24.4–76.7]	66.7 [42.8–90.5]
2 y	54.6 [42.1–67.1]	52.8 [37.7–67.9]	60.0 [17.1–100]	77.4 [47.5–100]	16.2 [0.0–42.4]	44.4 [18.5–70.4]

atezo, atezolizumab; bev; bevacizumab; CI, confidence interval; CPI, checkpoint inhibitor; CRC, colorectal cancer; IFNα, interferon-α; PEG, polyethylene glycol; NE, no statistic is created for a non-convergent model; NSCLC, non-small cell lung cancer; RCC, renal cell carcinoma. ^a^ Censored observation.

## Data Availability

Qualified researchers may request access to individual patient level data through the clinical study data request platform (https://vivli.org/). Further details on Roche’s criteria for eligible studies are available here (https://vivli.org/members/ourmembers/). For further details on Roche’s Global Policy on the Sharing of Clinical Information and how to request access to related clinical study documents, see here (https://www.roche.com/research_and_development/who_we_are_how_we_work/clinical_trials/our_commitment_to_data_sharing.htm).

## Supplementary Materials

The following are available online at https://www.mdpi.com/article/10.3390/curroncol28060455/s1, Figure S1: Dosing schedule for atezolizumab and interferon α-2b in dose-escalation stage of Arm B; Table S1: Inclusion and exclusion criteria for Arms B–D; Table S2: Reasons for study discontinuation; Table S3: Baseline tumor histology in patients with RCC; Table S4: Treatment Exposure in Arms B and C; Table S5: Most common (≥2%) treatment-emergent Grade ≥3 AEs in patients in Arm B; Table S6: Efficacy summary in patients with RCC; Table S7: Treatment-emergent Grade ≥3 AEs in patients in Arm C; Table S8: Treatment exposure in Arm D; Table S9: Most common (≥2%) treatment-emergent Grade ≥3 AEs in patients in Arm D, Supplemental Material and Methods.
