# Supplementary material for: Phase Ib Study of Atezolizumab Plus Interferon-α with or without Bevacizumab in Patients with Metastatic Renal Cell Carcinoma and Other Solid Tumors
Source: Curr Oncol. 2021 Dec 20;28(6):5466–79. doi: 10.3390/curroncol28060455 (PMC8700717; doi:10.3390/curroncol28060455)
Supplement: Supplementary file 1 [file curroncol-28-00455-s001.zip › curroncol-1488941-supplementary.pdf]

Supplementary Tables, Figures and Methods

Supplementary Table S1. Inclusion and exclusion criteria for Arms B-D.

Inclusion	Exclusion
**Overall Key Criteria**	
• ≥18 years<br>• Ability to comply with the study protocol<br>• Histologically/cytologically documented advanced/metastatic solid tumors<br>• ECOG performance status score of 0 or 1<br>• Life expectancy ≥12 weeks<br>• Measurable disease by RECIST v1.1<br>• Adequate hematologic and end organ function<br>• Agreement to remain abstinent or use contraceptive measures<br>• Archival tumor tissue	• NSCLC with sensitizing mutation in *EGFR* or *ALK* rearrangements<br>• Melanoma with *BRAF* mutations<br>• Active/untreated CNS metastases<br>• Spinal cord compression not definitively treated with surgery and/or radiation<br>• Leptomeningeal disease<br>• Uncontrolled pleural effusion, pericardial effusion, or ascites requiring recurrent drainage procedures<br>• Uncontrolled tumor-related pain<br>• Uncontrolled hypercalcemia<br>• History of other malignancy within 2 year prior to screening<br>• Pregnant and lactating women<br>• Any approved cancer therapy within 3 weeks prior to initiation of study treatment<br>• History of severe allergic reaction to chimeric or humanized antibodies or fusion proteins<br>• History of active autoimmune disease, idiopathic pulmonary fibrosis, or HIV<br>• Active hepatitis B, hepatitis C, or tuberculosis
**Criteria Specific to Arm B: Atezo + IFNα**	
• Disease progression during or after ≥1 previous systemic, anti-cancer treatment for locally advanced or metastatic NSCLC and RCC<br>• Dose escalation stage: Patients with RCC or melanoma<br>• Expansion stage: Patients with RCC or melanoma	• History of depression, suicidal ideation or behavior, bipolar disorder, or psychosis<br>• Hypersensitivity to interferon-alpha or any component of the product

• Mandatory biopsy cohort: Patients with RCC or melanoma<br>• Prior immunotherapy cohort: patients with RCC, NSCLC, or melanoma previously treated with PD-L1/PD-1	
**Criteria Specific to Arm C: Atezo + PEG-IFNα**	
• Disease progression during or after ≥1 previous systemic, anti-cancer treatment for locally advanced or metastatic NSCLC and RCC<br>• Cohort 1: Patients with RCC	• History of depression, suicidal ideation or behavior, bipolar disorder, or psychosis<br>• Hypersensitivity to interferon-alpha or any component of the product
**Criteria Specific to Arm D: Atezo + PEG-IFNα + Bev**	
• Cohort 1: Patients with metastatic RCC with no prior line of systemic therapy for metastatic disease<br>• Cohorts 2-3: Disease progression during of after ≥1 previous systemic, anti-cancer treatment for locally advanced/metastatic solid tumors<br>o Cohort 2: Patients with locally advanced/metastatic ns-NSCLC or CRC<br>o Cohort 3: Patients who were previously treated with anti-PD-L1/PD-1 with locally advanced/metastatic solid tumors<br>▪ Patients with melanoma who were previously treated with anti-CTLA-4 therapy could also be enrolled	• History of depression, suicidal ideation or behavior, bipolar disorder, or psychosis<br>• Hypersensitivity to interferon-alpha or any component of the product<br>• Inadequately controlled hypertension<br>• Prior history of hypotensive crisis<br>• Significant vascular disease within 6 months<br>• History of hemoptysis within 1 month<br>• Evidence of bleeding diathesis or significant coagulopathy<br>• Current/recent use of aspirin or parental anticoagulants of thrombolytic agents<br>• Core biopsy or other minor surgical procedures<br>• History of tracheoesophageal fistula, GI perforation, or intra-abdominal abscess within 6 months<br>• Clinical signs of GI obstruction<br>• Evidence of abdominal free air<br>• Serious, non-healing wound, active ulcer, or untreated bone fracture<br>• Proteinuria<br>• Metastatic disease that involves major airways or blood vessels<br>• Clear tumor infiltration into the thoracic great vessels<br>• Clear cavitation of pulmonary lesions

Supplementary Table S2. Reasons for study discontinuation.

Reason, *n* (%)	Arm B Atezo + IFNα (*n*=65)	Arm C Atezo + PEG-IFNα (*n*=6)	Arm D Atezo + PEG-IFNα + Bev		
			Cohort 1: 1L RCC (*n*=15)	Cohort 2: 2L+ CRC/NSCLC (*n*=15)	Cohort 3: prior CPI (*n*=15)
Death	40 (61.5)	2 (33.3)	2 (13.3)	10 (66.7)	8 (53.3)
Loss to follow-up	2 (3.1)	0	1 (6.7)	0	0
Withdrawal by subject	3 (4.6)	1 (16.7)	0	1 (6.7)	1 (6.7)
Study terminated by sponsor	3 (4.6)[a]	0	4 (26.7)	0	1 (6.7)
Other	17 (26.2)	3 (50.0)	7 (46.7)	4 (26.7)	4 (26.7)
Progressive disease	0	0	1 (6.7)	0	1 (6.7)

[a]Includes 1 patient who was entered as still on study in survival follow up, but study had been terminated by sponsor.

Supplementary Table S3. Baseline tumor histology in patients with RCC.

Characteristic	**Arm B** Atezo + IFNα		**Arm C** Atezo + PEG-IFNα	**Arm D** Atezo + PEG- IFNα + Bev	
	**CPI-naive RCC (*n*=45)**	**Prior CPI RCC (*n*=5)**	**2L+ RCC (*n*=6)**	**Cohort 1: 1L RCC (*n*=15)**	**Cohort 3: prior CPI RCC (*n*=6)**
**Tumor histology, *n* (%)**					
Clear cell	40 (88.9)	5 (100)	5 (83.3)	10 (66.7)	6 (100)
Chromophobe	1 (2.2)	0	0	1 (6.7)	0
Papillary	1 (2.2)	0	0	2 (13.3)	0
Sarcomatoid	0	0	0	1 (6.7)	0
Other	1 (2.2)	0	1 (16.7)	1 (6.7)	0
Missing	2 (4.4)	0	0	0	0

Supplementary Table S4. Treatment exposure in Arms B and C.

	**Arm B** Atezo + IFNα (*n*=65)		**Arm C** Atezo + PEG-IFNα (*n*=6)	
	**Atezo**	**IFNα**	**Atezo**	**PEG-IFNα**
Treatment duration, *n* (%), mo				
Median (range)	5.0 (0-59)	3.2 (0-9)	5.2 (1-26)	2.8 (1-3)
Mean (SD)	11.5 (13.9)	4.4 (3.4)	8.3 (9.8)	2.3 (1.4)
Treatment duration, *n* (%)				
1 to <3 mo	18 (27.7)	20 (30.8)	3 (50.0)	3 (50.0)
3 to <6 mo	16 (24.6)	23 (35.4)	0	3 (50.0)
6 to <9 mo	8 (12.3)	19 (29.2)	1 (16.7)	–
9 to <12 mo	4 (6.2)	3 (4.6)	1 (16.7)	–
≥12 mo	19 (29.2)	–	1 (16.7)	–
Number of doses				
Median (range)	7.0 (1-84)	9.0 (5-15)	8.5 (2-38)	5.0 (2-6)
Mean (SD)	16.9 (19.7)	10.1 (3.7)	12.8 (13.9)	4.3 (2.0)

Supplementary Table S5. Most common (≥2%) treatment-emergent Grade ≥3 AEs in patients in Arm B.

**AE, *n* (%)**	**Arm B**<br>**Atezo + IFNα**<br>**(*n*=65)**
Any AE	25 (38.5)
Pneumonia	5 (7.7)
Anemia	5 (7.7)
Lipase increased	4 (6.2)
Abdominal pain	3 (4.6)
Vomiting	3 (4.6)
Hyperglycemia	3 (4.6)
Acute kidney injury	3 (4.6)
Hematochezia	2 (3.1)
Nausea	2 (3.1)
Small intestinal obstruction	2 (3.1)
Amylase increased	2 (3.1)
Dyspnea	2 (3.1)
Pain in extremity	2 (3.1)

Supplementary Table S6. Efficacy summary in patients with RCC.

Characteristic	Arm B<br>Atezo + IFNα		Arm C<br>Atezo + PEG-IFNα	Arm D<br>Atezo + PEG-IFNα + Bev	
	CPI-naive RCC (*n*=45)	Prior CPI RCC (*n*=5)	2L+ RCC (*n*=6)	Cohort 1: 1L RCC (*n*=15)	Cohort 3: prior CPI RCC (*n*=6)
**Response, *n* (%) [95% CI]**					
Objective response rate	8 (17.8)<br>[6.6-29.0]	1 (20.0)<br>[0.0-55.1]	0<br>[0.0-0.0]	7 (46.7)<br>[21.4-71.9]	1 (16.7)<br>[0.0-46.5]
Complete response	1 (2.2)<br>[0.0-6.5]	0<br>[0.0-0.0]	0<br>[0.0-0.0]	1 (6.7)<br>[0.0-19.3]	0<br>[0.0-0.0]
Partial response	7 (15.6)<br>[5.0-26.1]	1 (20.0)<br>[0.0-55.1]	0<br>[0.0-0.0]	6 (40.0)<br>[15.2-64.8]	1 (16.7)<br>[0.0-46.5]
Stable disease	20 (44.4)<br>[29.9-59.0]	3 (60.0)<br>[17.1-100]	2 (33.3)<br>[0.0-71.0]	7 (46.7)<br>[21.4-71.9]	4 (66.7)<br>[29.0-100]
Progressive disease	16 (35.6)<br>[21.6-49.5]	1 (20.0)<br>[0.0-55.1]	3 (50.0)<br>[10.0-90.0]	1 (6.7)<br>[0.0-19.3]	1 (16.7)<br>[0.0-46.5]
Missing/unevaluable	1 (2.2)	0	1 (16.7)	0	0
**Duration of response, *n***	8	1	0	7	1
Median [95% CI], mo	24.9 [3.7-NE]	28.8 [NE]	–	12.5 [4.5-NE]	NE [NE]
Range	2.8-50.5[a]	28.8-28.8	–	2.8-25.8[a]	19.4[a]-19.4[a]
**Progression-free survival**					
Patients with event, *n* (%)	41 (91.1)	5 (100)	6 (100)	10 (66.7)	3 (50.0)
Median [95% CI], mo	3.2 [2.8-5.5]	5.8 [1.5-33.2]	1.9 [1.2-4.2]	9.0 [4.1-NE]	NE [4.0-NE]
Range	1-52[a]	2-33	1-9	1-28[a]	1-28[a]

Landmark rate [95% CI], %					
6 mo	33.3 [19.6-47.1]	40.0 [0.0-82.9]	16.7 [0.0-46.5]	71.8 [48.3-95.3]	66.7 [29.0-100]
1 y	20.0 [8.3-31.7]	40.0 [0.0-82.9]	NE	43.1 [17.1-69.0]	50.0 [10.0-90.0]
2 y	10.4 [1.2-19.6]	20.0 [0.0-55.1]	NE	28.7 [5.0-52.4]	50.0 [10.0-90.0]
**Overall survival**					
Patients with event, *n* (%)	30 (66.7)	2 (40.0)	2 (33.3)	2 (13.3)	2 (33.3)
Median [95% CI], mo	26.3 [15.6-37.6]	NE [5.4-NE]	NE [2.4-NE]	NE [17.7-NE]	NE [12.7-NE}
Range	3[a]-53[a]	5-46[a]	1[a]-26[a]	5[a]-30[a]	2-28[a]
Landmark rate [95% CI], %					
6 mo	93.1 [85.5-100]	80.0 [44.9-100]	80.0 [44.9-100]	100 [100-100]	83.3 [53.5-100]
1 y	74.4 [61.3-87.5]	80.0 [44.9-100]	80.0 [44.9-100]	92.9 [79.4-100]	83.3 [53.5-100]
2 y	52.8 [37.7-67.9]	60.0 [17.1-100]	60.0 [17.1-100]	77.4 [47.5-100]	66.7 [29.0-100]

Supplementary Table S7. Treatment-emergent Grade ≥3 AEs in patients in Arm C.

**AE, *n* (%)**	**Arm C**<br>**Atezo + PEG-IFNα**<br>**(*n*=6)**
Any adverse event	3 (50.0)
Blood creatinine increased	1 (16.7)
Constipation	1 (16.7)
Gastrointestinal hemorrhage	1 (16.7)
Anemia	1 (16.7)
Shock	1 (16.7)
Acute respiratory failure	1 (16.7)
Cough	1 (16.7)
Hypoxia	1 (16.7)
Sepsis	1 (16.7)
Hematuria	1 (16.7)
Muscular weakness	1 (16.7)
Cholangitis	1 (16.7)
Eyelid ptosis	1 (16.7)
Myasthenia gravis	1 (16.7)

Supplementary Table S8. Treatment exposure in Arm D.

Treatment characteristic, *n* (%)	Arm D<br>Atezo + PEG-IFNα + Bev<br>(*n*=45)								
	Cohort 1: 1L RCC<br>(*n*=15)			Cohort 2: 2L+ CRC/NSCLC<br>(*n*=15)			Cohort 3: prior CPI<br>(*n*=15)		
	Atezo	PEG-IFNα	Bev	Atezo	PEG-IFNα	Bev	Atezo	PEG-IFNα	Bev
Treatment duration, mo									
Median (range)	10.6 (2-30)	3.5 (2-4)	7.9 (2-28)	3.5 (1-26)	3.4 (1-4)	3.4 (1-26)	5.6 (0-28)	3.5 (0-4)	3.7 (0-28)
Mean (SD)	12.5 (9.0)	3.4 (0.4)	11.3 (7.9)	6.7 (7.3)	2.5 (1.2)	5.9 (7.2)	7.6 (7.5)	3.0 (1.1)	6.9 (7.5)
Treatment duration, *n* (%)									
1 to <3 mo	1 (6.7)	1 (6.7)	2 (13.3)	6 (40.0)	7 (46.7)	7 (46.7)	3 (20.0)	4 (26.7)	5 (33.3)
3 to <6 mo	3 (20.0)	14 (93.3)	2 (13.3)	4 (26.7)	8 (53.3)	4 (26.7)	5 (33.3)	11 (73.3)	4 (26.7)
6 to <9 mo	3 (20.0)	–	4 (26.7)	1 (6.7)	–	0	4 (26.7)	–	3 (20.0)
9 to <12 mo	2 (13.3)	–	1 (6.7)	1 (6.7)	–	1 (6.7)	1 (6.7)	–	1 (6.7)
≥12 mo	6 (40.0)	–	6 (40.0)	3 (20.0)	–	3 (20.0)	2 (13.3)	–	2 (13.3)
Number of doses									
Median (range)	15.0 (4-44)	6.0 (4-6)	10.0 (4-39)	6.0 (2-37)	5.0 (2-6)	4.0 (2-37)	9.0 (1-41)	6.0 (1-6)	6.0 (1-40)
Mean (SD)	18.7 (12.9)	5.8 (0.6)	15.2 (10.5)	10.3 (10.3)	4.4 (1.7)	8.5 (9.5)	11.8 (10.8)	5.2 (1.6)	10.5 (10.5)

Supplementary Table S9. Most common (≥2%) treatment-emergent Grade ≥3 AEs in patients in Arm D.

**AE, *n* (%)**	**Arm D**<br>**Atezo + PEG-IFNα + Bev**<br>**(*n*=45)**
Any AE	28 (62.2)
Hypertension	6 (13.3)
Lipase increased	4 (8.9)
Abdominal pain	3 (6.7)
Proteinuria	3 (6.7)
Urinary tract infection	2 (4.4)
Alanine aminotransferase increased	2 (4.4)
Blood alkaline phosphatase increased	2 (4.4)
Hypercalcemia	2 (4.4)
Lymphocyte count decreased	2 (4.4)
Nausea	2 (4.4)

Supplementary Figure S1. Dosing schedule for atezolizumab and interferon α-2b in dose-escalation stage of Arm B. Arrows indicate interferon α-2b (red arrows) and atezolizumab (black arrows) dosing at the indicated time points.

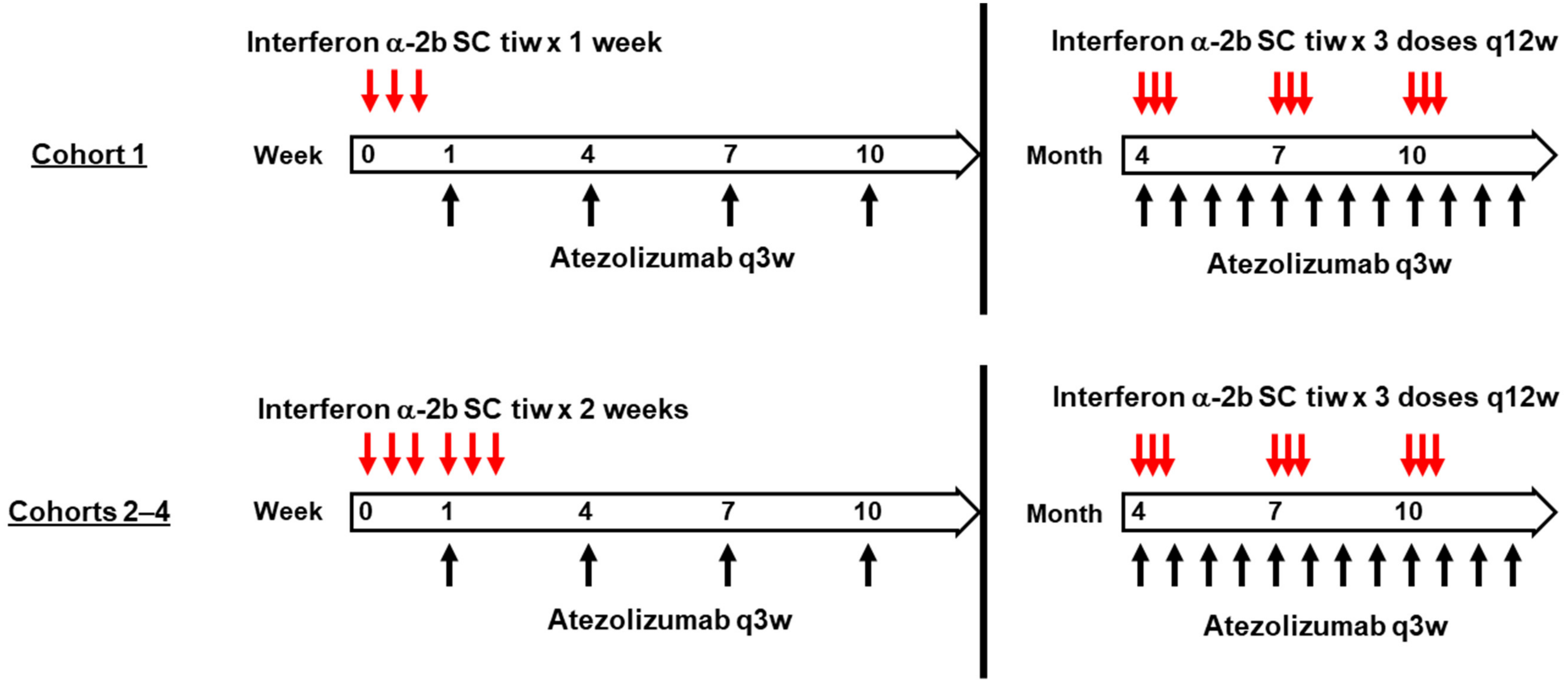

Supplemental Material and Methods:

Study Design

The dose-escalation stage of arm B was planned to include 15 to 29 patients to determine the recommended Phase II dose for atezolizumab combined with interferon α-2b using a standard 3 + 3 dose escalation. The expansion stage aimed to enroll approximately 20 patients with RCC and up to 10 with melanoma. As many as 10 more patients were planned in a separate biopsy expansion cohort, plus an additional cohort of up to 10 patients with prior anti–PD-L1/PD-1 treatment. Arm D was planned to include approximately 45 patients across 3 cohorts defined by tumor type and prior treatment status. Study conduct was in alignment with the Good Clinical Practice guidelines and the principles of the Declaration of Helsinki, and the study protocol was approved by independent ethics committees or institutional review boards at all study centers. All patients were required to provide written informed consent.

Patients

Arm B recruited patients with either melanoma or RCC, and disease progression after at least 1 prior line of systemic therapy, to the dose-escalation and -expansion stages. A further cohort in the dose-expansion stage of this arm enrolled patients with RCC, NSCLC, or melanoma who had previously received anti–PD-L1/PD-1 treatment.

Arm C recruited patients with RCC who had experienced disease progression after at least 1 prior line of systemic therapy. In arm D, patients in cohort 1 had RCC and no prior systemic therapy for metastatic disease. Cohorts 2 (NSCLC or colorectal carcinoma [CRC]) and 3 (RCC, NSCLC or melanoma) must have had at least 1 line of systemic therapy. Prior CPI treatment was required in cohort 3. Specific additional

exclusion criteria for arm D included inadequately controlled hypertension, a history of hemoptysis, significant coagulopathy or current therapeutic anticoagulation, and any minor surgical procedure within 7 days prior to the first bevacizumab dose.

Patients who had prior CPI treatment were generally ineligible for arms B, C, and D, except in specific cohorts that required prior CPI treatment. Additionally, patients were ineligible for study arms B, C, and D if they had a history of depression, suicidal ideation or behavior, bipolar disorder or psychosis, or if they were sensitive to interferon-α or its components.

Treatment

In arm B, cohort 1 started with interferon α-2b administered subcutaneously (SC) at 3 million international units (MIU), on days 1, 3, and 5 of cycle 1 only. Atezolizumab was dosed intravenous (IV) at 600 mg on day 8 of cycle 1 and day 1 of subsequent 21-day cycles. Cohorts 2-4 had interferon α-2b SC dosing at 3, 5, or 10 MIU on days 1, 3, 5, 8, 10, and 12 of cycle 1, with IV atezolizumab dosing at 1,200 mg on day 8 of cycle 1 and day 1 of subsequent cycles. Further interferon α-2b was given on days 1, 3, and 5 of cycles 5, 9, and 13 in all cohorts. The first treatment cycle incorporated a 1-week run-in period of interferon α-2b followed by atezolizumab dosing, and therefore comprised 28 days in all cohorts.

Endpoints and Assessments

Response and disease progression-based endpoints were investigator-assessed using RECIST 1.1. Tumor assessments were performed by computed tomography at screening and every 6 ($\pm$1) weeks for 48 weeks, then every 12 ($\pm$1) weeks, until death, radiographic disease progression, withdrawal of consent, commencement of a new anti-

cancer therapy, or study closure. Follow-up continued at 6-week intervals for patients receiving atezolizumab beyond disease progression. PD-L1 expression on tumor samples was determined using the VENTANA SP142 PD-L1 immunohistochemistry assay.

Statistical Analysis

The safety-evaluable population, defined as all patients who received any amount of any study drug, was used for all analyses. Sample sizes in each cohort were selected to obtain the required safety, pharmacokinetics, and pharmacodynamic information, as opposed to being determined for study power and type I error purposes.

Summary of continuous variables was performed using means, standard deviations, medians, and ranges, while categorical variables were summarized using counts and percentages. The ORR and its 95% confidence interval (CI) were estimated. DOR, the time from the initial response to the first to occur of disease progression or death, was determined for all patients achieving a confirmed objective response.

PFS was defined as the time from the first dose of study therapy to disease progression or death, whichever occurred first. OS was defined as the time from the first dose of study therapy to death from any cause. Kaplan-Meier curves were used to summarize time-to-event data.
